# Nursing Affairs in Ireland

**Published:** 1917-08-04

**Authors:** 


*To the Editor of* The Hospital.

### Nursing Affairs in Ireland.

*To the Editor of* The Hospital.

Sir,—If the meeting held at Belfast last week had nothing else to its credit, it has at least demonstrated that the Irish branch of the College of Nursing has found a jewel in its secretary, Miss Vera Matheson, whose address [see page 366] is a masterpiece of simplicity and comprehensiveness. This is by a long way the clearest and most convincing exposition of the aims and outlook of the College that has yet been put forward in Ireland. The possible advantages of membership are set out in relative proportion, and attention is drawn to the magnitude of the mistake made by the opponents of the College in supposing and teaching that State Registration and the Nursing Millennium are synonymous terms. Similarly the V. A. D. supremacy red-herring is ably dealt with. Throughout the address there is no trace of any note of bitterness, but on the contrary there is manifest a kindly and sympathetic attitude in every respect suited to an institution whose boundaries and whose outlook are Imperial. This address, I suggest, deserves a wide circulation in Great Britain as well as here. Miss Matheson is a brilliant scholar as well as a fully trained nurse, and the simple directness of her appeal is calculated to convince the rank and file of a profession too busily occupied to study Parliamentary Bills or arguments concerning technicalities which are usually full of sound and fury, and signify very little that is profitable.

The Irish Nursing Board, being the newly constructed Society whose *raison d'être* is opposition to the College, is busily occupied manufacturing bye-laws—the latest proposition being to raise the "registration" fee from one to two guineas. This is regarded by many within the fold as preposterous and unbusinesslike, and, naturally, the inquiry is made—what is to become of the guineas? Their ultimate disposal is, so far, shrouded in mystery, but, of course, the money is intended to pay fees to what they call in legal circles "investigators of title," or in other words to those persons whose duty it will be to certify the fitness of candidates for enrolment. If I may suggest it, however, such inquiries at this stage are premature. The guineas in question are still in the pockets of the nurses, and there they are likely to remain. These ladies were originally asked to pay a guinea, and they are now invited to pay twice this sum, and for what? In order that they may have granted to them a new certificate in lieu of the hospital certificate which they already possess. It does not appear to have occurred to them that an official certificate of effectual training may be obtained from the Local Government Board by any nurse who wishes to add to the force or authority of her hospital diploma.

Miss Matheson's timely address will go far to clear the air and to dissipate misunderstandings. Erroneous rumours are being circulated in nursing circles here to

(*Continued on p.* vi.)

